# Anti-Melanogenesis and Anti-Photoaging Effects of the Sulfated Polysaccharides Isolated from the Brown Seaweed *Padina boryana*

**DOI:** 10.3390/polym15163382

**Published:** 2023-08-11

**Authors:** Lei Wang, Thilina U. Jayawardena, Young-Sang Kim, Kaiqiang Wang, Xiaoting Fu, Ginnae Ahn, Seon-Heui Cha, Jeong Gyun Kim, Jung Suck Lee, You-Jin Jeon

**Affiliations:** 1 College of Food Science and Engineering, Ocean University of China, Qingdao 266003, China; 2 Department of Chemistry, Biochemistry and Physics, Université du Québec à Trois-Rivières, Trois-Rivières, QC G8Z 4M3, Canada; tuduwaka@gmail.com; 3 Department of Marine Life Sciences, Jeju National University, Jeju 63243, Republic of Korea; 4 Department of Marine Bio Food Science, Chonnam National University, Yeosu, 59626, Republic of Korea; 5 Department of Marine Bio and Medical Science, Hanseo Universirty, Seosan-si 32158, Republic of Korea; 6 Department of Seafood Science & Technology, Institute of Marine Industry, Gyeongsang National University, Tongyeong 53064, Republic of Korea; kimjg@gnu.ac.kr (J.G.K.); jungsucklee@hanmail.net (J.S.L.); 7 Marine Science Institute, Jeju National University, Jeju 63333, Republic of Korea

**Keywords:** *Padina boryana*, sulfated polysaccharides, melanogenesis, UVB irradiation

## Abstract

Sulfated polysaccharides isolated from seaweeds are thought of as ideal ingredients in the pharmaceutical, nutraceutical, and cosmetics industries. Our previous study isolated and characterized sulfated polysaccharides from *Padina boryana*. The sulfated polysaccharides of *Padina boryana* (PBP) were extracted, and the antioxidant activity of PBP was evaluated. The results indicate that PBP possesses antioxidant effects and potential in the cosmetic industry. To further investigate the potential of PBP in cosmetics, the photoprotective and anti-melanogenesis effects of PBP were evaluated. The anti-melanogenesis test results display that PBP reduced the melanin content in the murine melanoma cells stimulated by alpha melanocyte-stimulating hormone from 203.7% to 183.64%, 144.63%, and 127.57% at concentrations of 25 μg/mL, 50 μg/mL, and 100 μg/mL, respectively. The anti-photodamage test results showed that PBP significantly protected skin cells against UVB-stimulated photodamage. PBP suppressed human epidermal keratinocyte (HaCaT cell) death by inhibiting apoptosis and reducing the level of intracellular reactive oxygen species. The intracellular reactive oxygen species level of HaCaT cells irradiated by UVB was reduced from 192.67% to 181.22%, 170.25%, and 160.48% by 25 μg/mL, 50 μg/mL, and 100 μg/mL PBP, respectively. In addition, PBP remarkably reduced UVB-induced human dermal fibroblast damage by suppressing oxidative damage, inhibiting collagen degradation, and attenuating inflammatory responses. These results indicate that PBP possesses photoprotective and anti-melanogenesis activities and suggest that PBP is a potential ingredient in the cosmetic industry.

## 1. Introduction

Human skin is continually damaged by environmental factors such as cigarette smoking, environmental chemicals, ultraviolet (UV) irradiation, and air pollution [1,2,3,4,5,6,7]. These environmental factors damage the skin through inflammatory responses, oxidative stress, collagen degradation, and pigment disorders [8,9,10]. The accumulation of this damage causes skin dysfunction and finally leads to skin aging and aging-related diseases. Skin is an unavoidable exposure to these environmental factors. Thus, it is necessary to take measures to protect the skin against damage from these environmental factors. Therefore, a skincare product that protects skin from damage has a huge market demand. Owing to the advantages of natural products such as efficiency and safety, discovering bioactive natural compounds and developing them into skincare products has attracted researchers’ attention.

Seaweeds are rich in natural compounds, including sterols, polyphenols, proteins, pigments, flavonoids, and polysaccharides [11,12,13,14,15,16,17,18]. Seaweed, in particular, contains high amounts of polysaccharides. For example, laminaran and alginate in brown seaweed, and carrageenan and agar in red seaweed [19,20,21,22,23,24]. Sulfated polysaccharides purified from brown seaweed have been reported with their various bioactivities, including anti-diabetic, anti-inflammatory, anti-obesity, anti-virus, anti-tumor, antioxidant, prebiotic, and UV protective effects [21,25,26,27,28,29,30,31,32,33]. Arunkumar et al. reported the antioxidant activities of five sulfated polysaccharides extracted from different edible seaweeds, including *Centroceras clavulatum* (CC), *Asparagopsis taxiformis* (AT), *Spyridia hypnoides* (SH), *Portieria hornemannii* (PH), and *Padina pavonica* (PP) [28]. The results showed that the sulfated polysaccharides isolated from CC, AT, SH, PH, and PP possess a DPPH radical scavenging effect and iron-reducing power [28]. Barbosa et al. evaluated the anti-tumor effect of sulfated polysaccharides isolated from *Caulerpa cupressoides* var. flabellate [32]. The results demonstrated that the sulfated polysaccharides from *Caulerpa cupressoides* var. flabellate effectively inhibited the proliferation of mouse melanoma cells (B16F10 cells) [32].

*Padina boryana* (*P. boryana*) is a popular brown seaweed in Asian countries. *P. boryana* is widely distributed in the Laccadive Sea. However, few reports concern the bioactivities of *P. boryan*. In previous studies, we isolated sulfated polysaccharides from *P. boryana* (PBP) and investigated the antioxidant activity of PBP in in vitro and in vivo models. The results indicated that PBP effectively suppressed oxidative stress in in vitro and in vivo models and suggested the potential of PBP in the cosmetic industry [34]. In order to further explore the cosmetic potential of PBP, the anti-photoprotective and melanogenesis effects of PBP were evaluated in the current study. The objectives were as follows: to evaluate the anti-melanogenesis effect of PBP in murine melanoma cells (B16F10 cells); to investigate the photoprotective effect of PBP in human epidermal keratinocytes (HaCaT cells) and human dermal fibroblasts (HDF cells); and to confirm the potential of PBP in the cosmetic industry.

## 2. Materials and Methods

### 2.1. Chemicals and Reagents

Mushroom tyrosinase, dimethyl sulfoxide, 2,7-dichlorofluorescein diacetate, and Hoechst 33342 were purchased from Sigma (St. Louis, MO, USA). Penicillin-streptomycin, Ham’s F-12 Nutrient Mixture, trypsin-EDTA, enzyme-linked immunosorbent assay kits, and Dulbecco’s Modified Eagle Medium were purchased from Gibco-BRL (Grand Island, NY, USA).

The seaweed, *P. boryana,* was harvested from the shores of Fulhadhoo Island, the Maldives, in August 2018. The seaweed was washed with tap water to remove the impurities and salt. The washed seaweed was lyophilized and crushed into powder. The seaweed powder was maintained at 4 °C until use.

### 2.2. Preparation and Characterization of Sulfated Polysaccharides from P. boryana

The sulfated polysaccharides from *P. boryana* (PBP) were prepared and characterized in the previous study [34]. In brief, the seaweed powder (50 g) was mixed with distilled water (0.5 L). The pH of the mixture was adjusted to 4.5 using hydrochloric acid (1 M). Then, optimal Celluclast-assisted extraction of the mixture was carried out over a period of 24 h under shaking conditions at 50 °C. After extraction, the Celluclast was inactivated by heating at 100 °C for 10 min. The pH was brought back to 7 using a sodium hydroxide solution (1 M). After filtration, the supernatant was added to a three-fold volume of 95% ethanol and maintained at 4 °C. After 12 h, The precipitate was collected, and the polysaccharides from *P. boryana* were obtained. The polysaccharides of *P. boryana* were designated as PBP.

The chemical composition of PBP, including total polyphenol content, polysaccharide content, and sulfate content, was analyzed using the method described by Chandler and Dodds, the official methods of AOAC analysis, and the barium chloride gelation method, respectively [34]. The results indicated that PBP contains 56.34% sulfated polysaccharides and 1.14% phenolic content.

The monosaccharide of PBP was analyzed. The PBP was hydrolyzed using 4 M triflouroacetic acid at 100 °C for 4 h. Then, the sample was subjected to the CarboPac PA1 cartridge column for separation and detected with an ED50 Dionex electrochemical detector. The monosaccharide analysis results indicated that the polysaccharides of PBP are comprised of fucose (57.51%), galactose (21.35%), mannose (13.21%), and others. In addition, the major functional group in the structure of PBP was characterized by FTIR [34].

### 2.3. Evaluation of Tyrosinase and Collagenase Inhibitory Effect of PBP

To investigate the tyrosinase inhibitory effect of PBP, a reaction mixture containing 10 μL of mushroom tyrosinase solution, 10 μL of PBP solution, 40 μL of *L*-tyrosine (1.5 mM), and 140 μL of phosphate buffer (50 mM, pH 6.5) in a 96-well plate was incubated at 37 °C for 12 min. Then, the 96-well plate was kept on ice to stop the reaction. After cooling, the total amount of dopachrome produced by the reaction was analyzed.

To evaluate the collagenase inhibitory activity of PBP, the 100 μL PBP solution was mixed with 800 μL of Tris-HCl (0.1 M, pH 7.0), a weight of 1 mg of azo dye-impregnated collagen, and 100 μL of collagenase. After mixing, the mixture was incubated under shaking conditions at 43 °C for 1 h. After reaction, the reacted mixture was centrifuged (3000 rpm) for 10 min. After cooling, the total amount of dopachrome produced by the reaction was analyzed.

### 2.4. Maintenance of Cell Lines

HaCaT cells (Korean Cell Line Bank, Seoul, Republic of Korea) were cultured and seeded at a dose of 1 × 10^5^ cells/mL for the experiments. B16F10 cells (ATCC ^®^ CRL 6475 ™, Manassas, VA, USA) were cultured and seeded at a dose of 5 × 10^4^ cells/mL for the experiments. HDF cells (Korean Cell Line Bank, Seoul, Republic of Korea) were cultured and seeded at a dose of 5 × 10^4^ cells/mL for the experiments.

### 2.5. Determination of Cytotoxicity of PBP

PBP was dissolved in 1× PBS buffer to make a stock solution for the experiments. Based on the previous report, PBP was non-toxic to Vero cells at concentrations of 25 μg/mL, 50 μg/mL, and 100 μg/mL [34]. In the current study, we confirm the cytotoxicity of the cell lines we used for these concentrations. The viability of the control group of cells treated with 1× PBS buffer instead of the sample solution was referred to as 100%. To evaluate the cytotoxicity of PBP, B16F10 cells, HaCaT cells, and HDF cells were seeded and treated with 25 μg/mL, 50 μg/mL, and 100 μg/mL PBP for 24 h, respectively. The viability of PBP-treated B16F10 cells, HaCaT cells, and HDF cells was evaluated using an MTT assay [34,35].

### 2.6. Determination of the Anti-Melanogenesis Activity of PBP

To measure the anti-melanogenesis effect of PBP, B16F10 cells were seeded in a 6-well plate and treated with PBP. After 1 h of incubation, the PBP-treated B16F10 cells were stimulated with 50 nM alpha melanocyte stimulating hormone (α-MSH) and incubated for 72 h. After incubation, the α-MSH-stimulated B16F10 cells were harvested and discussed in sodium hydroxide (1 N) containing 10% dimethyl sulfoxide at 80 °C for 1 h. Then, the solution was centrifuged (13,000 g) for 10 min. The absorbance of the supernatant was measured using a microplate reader (BioTek, Synergy, UT, USA) at a wavelength of 490 nm. The melanin content of the control group was 100%.

### 2.7. Determination of the Photoprotective Effect of PBP on HaCaT Cells

To evaluate the photoprotective effect of PBP on HaCaT cells, HaCaT cells were seeded in a 24-well plate. After 24 h of incubation, HaCaT cells were treated with PBP for 1 h. The PBP-treated HaCaT cells were irradiated with UVB (30 mJ/cm^2^). To evaluate the intracellular ROS level of HaCaT cells, the UVB-irradiated HaCaT cells were treated with 2,7-dichlorofluorescein diacetate (500 μg/mL, 25 μL/well) for 30 min, and then the fluorescence intensities of HaCaT cells were evaluated. To evaluate the cell viability, the UVB-irradiated HaCaT cells were further incubated for 24 h. After incubation, the MTT solution (2 mg/mL, 25 μL/well) was added to each well, and the cells were incubated for 3 h. Then, the supernatant was removed, and the formazan was dissolved in dimethyl sulfoxide. The apoptosis body formation of UVB-irradiated HaCaT cells was investigated by the Hoechst 33343 assay [34]. The intracellular ROS level and cell viability of the control group were referred to as 100%.

### 2.8. Determination of the Photoprotective Effect of PBP in HDF Cells

HDF cells were seeded in a 24-well plate for 24 h and treated with PBP for 1 h. PBP-treated HDF cells were irradiated with UVB (50 mJ/cm^2^). UVB irradiation was carried out using a UVB meter equipped with a fluorescent bulb emitting 280~320 nm wavelength with a peak at 313 nm. Then, the viability and intracellular ROS levels of UVB-irradiated HDF cells were evaluated by MTT and 2,7-dichlorofluorescein diacetate assays, respectively [12]. The pro-inflammatory cytokines, collagen, and matrix metalloproteinase levels of the UVB-irradiated HDF cells were evaluated by enzyme-linked immunosorbent assay [12,36]. The cell viability, pro-inflammatory cytokines, collagen, intracellular ROS, and matrix metalloproteinases levels of the control group were referred to as 100%.

### 2.9. Statistical Analysis

All the experiments were conducted in triplicate in this study. The data were expressed as the mean ± standard error (SE), and a one-way ANOVA was used to compare the mean values using SPSS 20.0. Significant differences between the means were identified by the Tukey test.

## 3. Results and Discussion

### 3.1. Anti-Melanogenesis Effect of PBP

Melanin is a pigment widely found in human hair, eyes, mucosa, gallbladder, and skin. It is the key pigment that determines the hair and skin color of humans [37,38,39]. Melanin plays an important role in human skin health. For example, melanin protects the skin from photoaging stimulated by UV irradiation. Increasing melanogenesis will lead to skin pigmentation conditions such as senile plaques, nevi, and erythema [40]. Inhibition of melanogenesis was thought of as a strategy to prevent hyperpigmentation and related skin problems.

**Figure 1:**
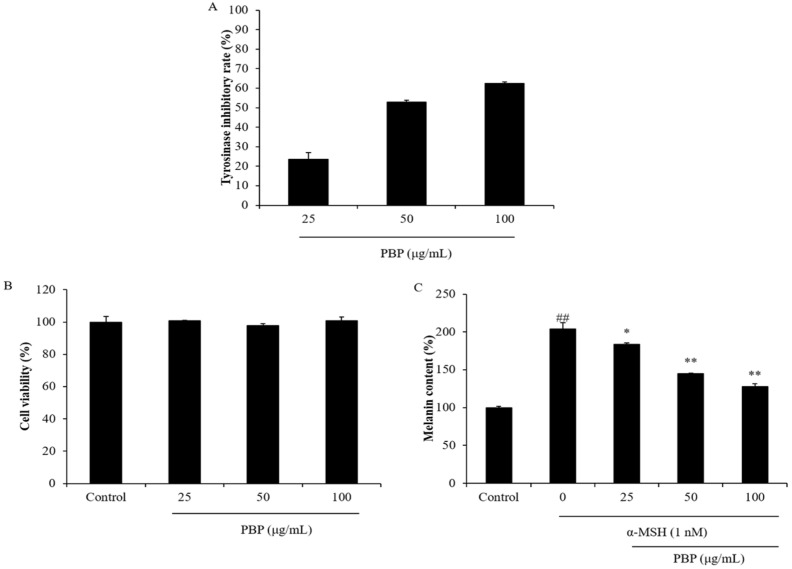
Skin whitening effect of PBP. (**A**) Tyrosinase inhibitory effect of PBP; (**B**) cytotoxicity of PBP on B16F10 cells; (**C**) inhibitory effect of PBP on α-MSH-stimulated melanin synthesis in B16F10 cells. The experiments were conducted in triplicate and the data are expressed as the mean ± SE. * p < 0.05, ** p < 0.01 as compared to the α-MSH-treated group and *## p* < 0.01 as compared to the control group.

Our previous study suggested that *P. boryana* possesses potential for anti-melanogenesis [34,41]. In the current research, the anti-melanogenesis effect of PBP was evaluated by investigating the inhibitory effect of PBP on tyrosinase from mushrooms and the melanin synthesis level in B16F10 cells. As Figure 1A shows, the PBP inhibits 23.57%, 52.86%, and 62.36% of tyrosinase at concentrations of 25 μg/mL, 50 μg/mL, and 100 μg/mL, respectively. This result showed that PBP concentration-dependently inhibited tyrosinase. It suggested the PBP possesses the potential for an anti-melanogenesis effect. Previous research demonstrated that the sulfated polysaccharides isolated from the brown seaweed *Sargassum fusiforme* inhibited 4.36%, 11.97%, and 36.66% of tyrosinase at concentrations of 25 μg/mL, 50 μg/mL, and 100 μg/mL, respectively [42]. In addition, a previous study showed that the sulfated polysaccharides isolated from the brown seaweed *Ecklonia maxima* showed 17.87%, 20.88%, and 26.31% tyrosinase inhibitory effects under concentrations of 25 μg/mL, 50 μg/mL, and 100 μg/mL, respectively [11]. Comparing the previous and current data, PBP possesses a stronger tyrosinase inhibitory effect than the sulfated polysaccharides isolated from the two brown seaweeds, *Sargassum fusiforme* and *Ecklonia maxima*.

Further study indicated that PBP effectively decreased melanin synthesis in the α-MSH-stimulated B16F10 cells, but was non-toxic to B16F10 cells (Figure 1B,C). As Figure 1C shows, the melanin synthesis level of B16F10 cells stimulated by α-MSH was doubled. However, the melanin contents of α-MSH-treated cells were decreased from 203.7% to 183.64%, 144.63%, and 127.57% by PBP at concentrations of 25 μg/mL, 50 μg/mL, and 100 μg/mL, respectively (Figure 1C). These data further confirm the anti-melanogenesis effect of PBP and suggest PBP may be a potential skin-whitening ingredient in the cosmetic industry.

### 3.2. Protective Effect of PBP on Photodamage Stimulated by UVB

UV irradiation is thought of as the primary environmental factor that causes skin aging. Overexposure to UV causes skin aging, and skin aging stimulated by UV irradiation is referred to as photoaging. UVB with a wavelength of 290–320 nm brings more stress to the skin compared to other types of UV [43]. UVB leads to epidermal and dermal damage by inducing intracellular ROS production [44]. Previous results demonstrated that PBP possesses ROS-scavenging activity and indicated its potential photoprotective effects. In order to investigate the photoprotective activity of PBP, the protective effect of PBP against UVB-stimulated skin damage was investigated in vitro in HaCaT cells and HDF cells.

**Figure 2:**
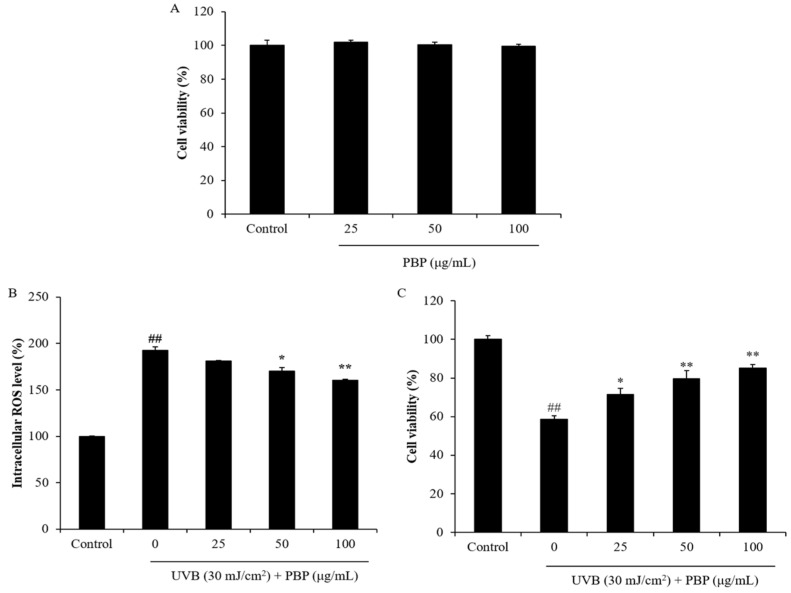
PBP protects HaCaT cells against UVB-induced photodamage. (**A**) Cytotoxicity of PBP on HaCaT cells; (**B**) intracellular ROS-scavenging effect of PBP; (**C**) protective effect of PBP against UVB-induced cell death. The experiments were conducted in triplicate and the data are expressed as the mean ± SE. * p < 0.05, ** p < 0.01 as compared to the UVB-irradiated group and *## p* < 0.01 as compared to the control group.

**Figure 3:**
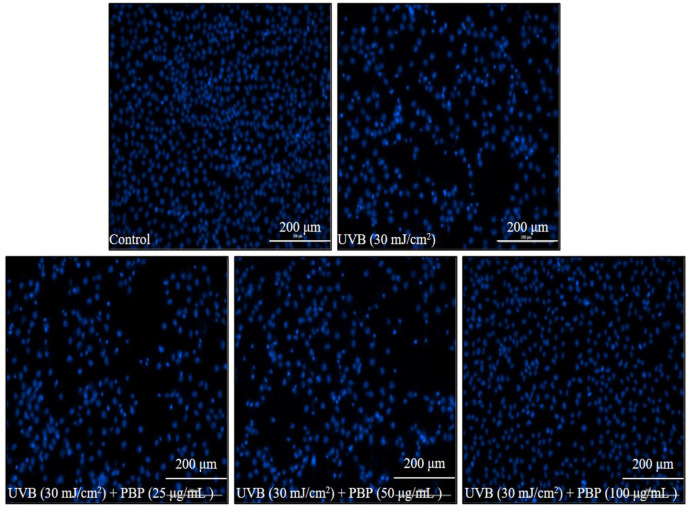
PBP protects HaCaT cells against UVB-induced apoptosis.

As Figure 2A shows, the viability of HaCaT cells treated with 25 μg/mL, 50 μg/mL, and 100 μg/mL PBP was not significantly decreased. These data indicated that PBP is non-toxic to HaCaT cells at these concentrations. Thus, these concentrations were applied to HaCaT cells in the further experiment, and the results are summarized in Figure 2B. As the results show, the intracellular ROS level of UVB-irradiated HaCaT cells increased from 100% (the control group) to 192.67%. However, the intracellular ROS levels of UVB-irradiated HaCaT cells were significantly reduced by PBP. The intracellular ROS levels of the cells treated with 25 μg/mL, 50 μg/mL, and 100 μg/mL PBP decreased from 192.67% to 181.22%, 170.25%, and 160.48%, respectively (Figure 2B). UVB significantly decreased the viability of HaCaT cells to 58.52% compared to the control group, whereas the viability of the HaCaT cells irradiated by UVB increased to 71.44%, 79.62%, and 85.12% using 25 μg/mL, 50 μg/mL, and 100 μg/mL PBP, respectively (Figure 2C). Further data display that PBP effectively attenuated apoptosis stimulated by UVB irradiation in HaCaT cells. The effect was shown in a concentration-dependent manner (Figure 3). These data demonstrate that PBP effectively protected HaCaT cells against cellular damage caused by UVB irradiation by scavenging intracellular ROS.

**Figure 4:**
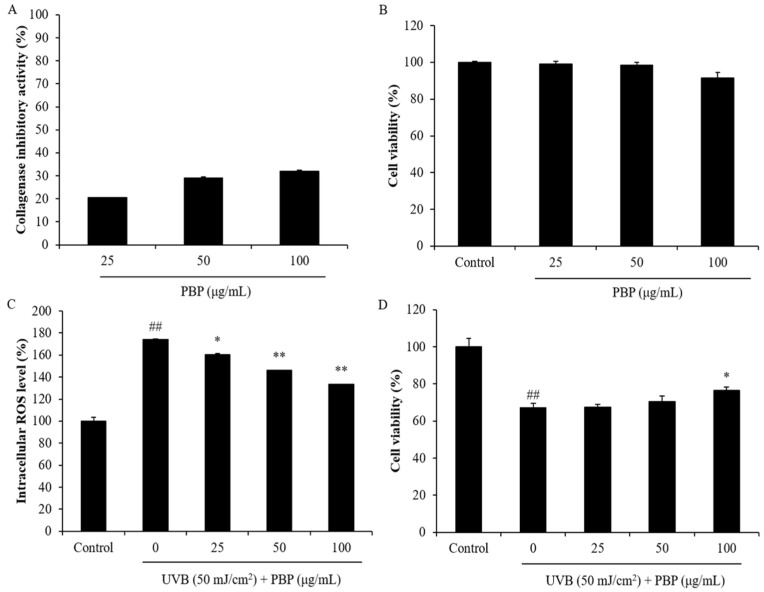
PBP inhibits collagenase and protects HDF cells against UVB-induced damage. (**A**) Collagenase inhibitory effect of PBP; (**B**) cytotoxicity of PBP on HDF cells; (**C**) intracellular ROS-scavenging effect of PBP; (**D**) protective effect of PBP against UVB-induced cell death. The experiments were conducted in triplicate and the data are expressed as the mean ± SE. * p < 0.05, ** p < 0.01 as compared to the UVB-irradiated group and *## p* < 0.01 as compared to the control group.

Collagen is one of the key structural and functional proteins in the skin, as well as the major component of the extracellular matrix. Collagen is degraded by different kinds of collagenases, and the degradation of collagen can cause skin thickness, which is one of the major characteristics of skin wrinkling. Thus, a collagenase inhibitor that can effectively inhibit the degradation of collagen may be a potential candidate to protect skin against wrinkling. The collagenase inhibitory effect of PBP was investigated, and the results are summarized in Figure 4A. As the results show, PBP inhibited 20.63%, 29.00%, and 32.06% of collagenase at concentrations of 25 μg/mL, 50 μg/mL, and 100 μg/mL, respectively (Figure 4A). This result indicates that PBP could inhibit collagenase and may be a potential candidate compound to protect skin against wrinkling.

In further experiments, the effect of PBP on the dermic damage caused by UVB irradiation was evaluated in HDF cells. As Figure 4B shows, PBP was non-toxic on HDF cells at a concentration below 100 μg/mL. Thus, the maximum concentration of PBP applied to HDF cells was decided to be 100 μg/mL. In addition, PBP significantly reduced the intracellular ROS levels of HDF cells irradiated by UVB from 174.13% to 160.36%, 146.33%, and 133.44% at concentrations of 25 μg/mL, 50 μg/mL, and 100 μg/mL, respectively (Figure 4C). UVB irradiation significantly decreased the viability of HDF cells (Figure 4D). However, the viability of HDF cells irradiated by UVB irradiation was increased by PBP (Figure 4D). These data indicate that PBP could protect HDF cells against photodamage stimulated by UVB irradiation.

**Figure 5:**
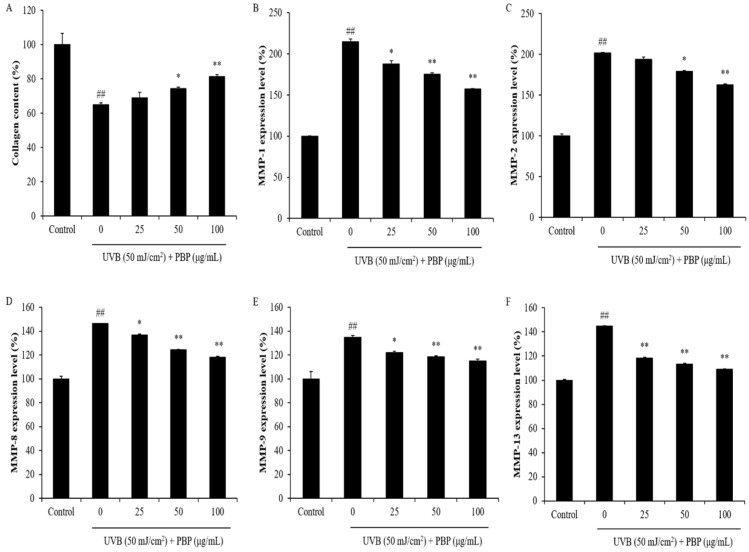
PBP increases collagen synthesis and inhibits MMP expression in UVB-irradiated HDF cells. (**A**) Collagen synthesis levels in UVB-irradiated HDF cells; (**B**) MMP-1 expression levels; (**C**) MMP-2 expression levels; (**D**) MMP-8 expression levels; (**E**) MMP-9 expression levels; (**F**) MMP-13 expression levels. The experiments were conducted in triplicate and the data are expressed as the mean ± SE. * p < 0.05, ** p < 0.01 as compared to the UVB-irradiated group and *## p* < 0.01 as compared to the control group.

**Figure 6:**
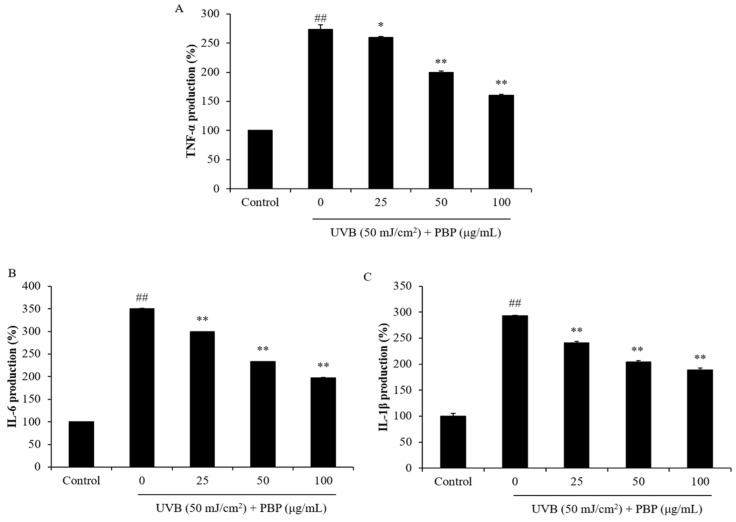
Inhibitory effects of PBP on the expression of pro-inflammatory cytokines in UVB-irradiated HDF cells. Expression of (**A**) TNF-α; (**B**) IL-6; and (**C**) IL-1β. The experiments were conducted in triplicate and the data are expressed as the mean ± SE. * p < 0.05, ** p < 0.01 as compared to the UVB-irradiated group and *## p* < 0.01 as compared to the control group.

MMPs are different kinds of collagenase that degrade different types of collagen. The collagen content and MMP production of UVB-irradiated HDF cells were evaluated. Compared to normal cells, the collagen content of UVB-irradiated HDF cells was reduced from 100% to 64.90%. However, the collagen content of UVB-irradiated HDF cells increased from 64.90% to 69.00%, 74.45%, and 81.43%, respectively (Figure 5A). In addition, the MMP levels of HDF cells significantly increased by UVB irradiation. However, the MMP expression of UVB-irradiated HDF cells was remarkably reduced by PBP (Figure 5B–F). These results demonstrate that PBP protects HDF cells against collagen degradation stimulated by UVB irradiation via inhibiting MMP expression. As shown in Figure 6, UVB irradiation stimulated pro-inflammatory cytokines, including tumor necrosis factors, interleukin (IL)-6, and IL-1β. However, the levels of these pro-inflammatory cytokines in UVB-irradiated HDF cells were significantly reduced in a concentration-dependent manner (Figure 6). These results indicated that PBP reduced inflammatory responses stimulated by UVB irradiation in HDF cells. Taken together, the above results demonstrate that PBP prevents dermal damage stimulated by UVB irradiation by suppressing oxidative stress, inhibiting collagen degradation, and reducing the expression of pro-inflammatory cytokines.

In summary, the present results indicate that PBP possesses anti-melanogenesis and photoprotective effects. The anti-melanogenesis effect is displayed by inhibiting tyrosinase activity and reducing melanin content in α-MSH-stimulated B16F10 cells. The photoprotective effect of PBP demonstrated that PBP effectively protected HaCaT cells against cellular damage caused by UVB irradiation by scavenging intracellular ROS and prevented HDF cells from damage stimulated by UVB irradiation by suppressing oxidative stress, inhibiting collagen degradation, and reducing the expression of pro-inflammatory cytokines. The present results suggest PBP may be a potential ingredient in the cosmetic industry. Previous studies suggest that the sulfated polysaccharides isolated from brown seaweeds possess a cosmetic effect, and the effect is related to the fucose content, sulfate content, molecular weight, and structure [11,42]. PBP possesses the above effect, which may be related to its fucose content. To further investigate the relationship between the structure and bioactivity of PBP, further purification and characterization are required.

## 4. Conclusions

In the present study, we investigated the anti-melanogenesis and photoprotective effects of PBP. The results indicate that PBP possesses the cosmetic potential demonstrated in reducing melanogenesis and inhibiting photodamage. The present study suggests that PBP could be used as an ingredient in a cosmetic to prevent skin aging. To develop PBP as a cosmetic to treat and prevent UVB-induced skin damage, the solubility, solution viscosity, pH stability, and other parameters, as well as the application of dosage forms and the clinical study of PBP, should be further studied.

## Data Availability

Data is contained within the article.
